# Effect of Different Medium-Chain Triglycerides on Glucose Metabolism in High-Fat-Diet Induced Obese Rats

**DOI:** 10.3390/foods13020241

**Published:** 2024-01-11

**Authors:** Jiaheng Xia, Zhixin Wang, Ping Yu, Xianghui Yan, Junxin Zhao, Guohua Zhang, Deming Gong, Zheling Zeng

**Affiliations:** 1 School of Chemistry and Chemical Engineering, Nanchang University, Nanchang 330031, China; xia_jheng@163.com (J.X.); zhixinwang2003@hotmail.com (Z.W.); 2 Jiangxi Province Key Laboratory of Edible and Medicinal Resources Exploitation, Nanchang University, Nanchang 330031, China; 3 School of Resources and Environment, Nanchang University, Nanchang 330031, China; xianghui_y@163.com; 4 School of Food Science and Technology, Nanchang University, Nanchang 330031, China; junxinzhao@hotmail.com; 5 Institute of Biological Resources, Jiangxi Academy of Sciences, Nanchang 330096, China; zhangguohua2050@163.com; 6 New Zealand Institute of Natural Medicine Research, 8 Ha Crescent, Auckland 2104, New Zealand; dgong01@gmail.com; 7 State Key Laboratory of Food Science and Resource, Nanchang University, Nanchang 330047, China

**Keywords:** medium-chain triglycerides, obesity, insulin resistance, PPARγ, hyperglycemia

## Abstract

Obesity can be associated with significant metabolic disorders. Our previous study found that medium-chain triglycerides (MCTs) improved lipid metabolism in obese rats. However, scant attention has been given to exploring the impact of MCTs on glucose metabolism in obese rats. This study is designed to examine the effects and mechanisms of three distinct MCTs on glucose metabolism in obese rats. To induce obesity, Sprague–Dawley (SD) rats were fed a high-fat diet, followed by a 12-week treatment with caprylic triglyceride (CYT), capric triglyceride (CT), and lauric triglyceride (LT). The results showed that three types of MCT intervention reduced the levels of lipids (TC, TG, LDL-c, and HDL-c), hyperglycemia, insulin resistance (insulin, OGTT, HOMA-IR, and ISI), and inflammatory markers (IL-4, IL-1β, and TNF-α) in obese rats (*p* < 0.01), The above parameters have been minimally improved in the high-fat restoring group (HR) group. MCTs can modulate the PI3K/AKT signaling pathways to alleviate insulin resistance and improve glucose metabolism in obese rats. Furthermore, MCTs can activate peroxisome proliferator-activated receptor (PPAR) γ and reduce the phosphorylation of PPARγ^ser237^ mediated by CDK5, which can improve insulin sensitivity without lipid deposition in obese rats. Among the MCT group, CT administration performed the best in the above pathways, with the lowest blood glucose level and insulin resistance. These findings contribute to a deeper understanding of the connection between health benefits and the specific type of MCT employed.

## 1. Introduction

Obesity has emerged as a global health concern, given its association with multiple metabolic disorders that increase the risk of metabolic comorbidities, like hepatic steatosis, cardiovascular disease, type 2 diabetes mellitus (T2DM), and even cancer [1]. T2DM is pathologically characterized by insulin resistance and early hyperinsulinemia, subsequently progressing to a decline in insulin production within pancreatic β cells [2]. The incidence of T2DM has increased along with the rising trend of obesity, and about 537 million people worldwide are reported to have diabetes, and this number is estimated to grow to 783 million by 2045, and nearly 90% of people with diabetes have T2DM [3].

The peroxisome proliferator-activated receptor (PPAR) γ, a ligand-activated nuclear receptor, has been reported to have a crucial role in maintaining lipid and glucose homeostasis via regulating the genes involved in lipid and glucose metabolism [4]. Hu et al. found that atorvastatin ester regulated hyperlipidemia through the PPAR-signaling pathway [5], while PPARγ activation has been shown to increased insulin sensitivity and enhance glucose metabolism in vivo [6]. Rosiglitazone and pioglitazone, members of the thiazolidinediones (TZDs), are classical drugs that have traditionally been used to treat insulin resistance in T2DM, and the effects of the two drugs have been mediated through binding the PPARγ and have acted as an agonist of PPARγ [7]. It was reported that activated PPARγ promoted the phosphorylation of phosphatidylinositol 3 kinase (PI3K), thereby activating the PI3K/AKT pathway [8]. Thus, a series of cascaded signals are activated, which can improve the insulin sensitivity, and the activated PI3K/AKT signal can also regulate gluconeogenesis and glycogen synthesis to maintain glucose homeostasis [9,10,11].

It has been reported that antidiabetic PPARγ agonists, including rosiglitazone, offer the ability to inhibit the phosphorylation of PPARγ at ser273 [12]. In the context of obesity, a high-fat diet activates cyclin-dependent kinase 5 (CDK5), leading to an upsurge in the phosphorylation of PPARγ at ser273. This event triggers the dysregulation of several insulin-sensitivity-related adipokines, such as adiponectin and adipsin [12,13]. However, rosiglitazone has been associated with certain side effects, including body weight gain, peripheral edema, and congestive heart failure [14,15]. These adverse effects have significantly limited the clinical use of thiazolidinediones (TZDs) in diabetes treatment. Consequently, extensive research endeavors have recently been initiated to investigate the potential of partial agonists or non-adipogenic agonists of PPARγ [16].

The medium-chain oils, which mainly consist of medium-chain triglycerides (MCTs), are known to have some health benefits in vivo. MCT treatment was shown to decrease food intake and BW gain, improve lipid metabolism, and induce thermogenic features both in the liver and the adipose tissue [17,18,19]. MCT intervention can also prevent postprandial hypertriglyceridemia in moderately hypertriglyceridemic patients [20] and ameliorate insulin resistance and hyperglycemia in vivo [21,22]. Moreover, in our previous work, MCT intervention increased the expression of PPARγ protein both in the liver and the adipose tissue in obese rats [23]. However, the mechanism by which MCTs improve glucose metabolism in obese rats is unclear. In addition, the effect of MCTs in improving triglyceride metabolism, cholesterol metabolism, and thermogenesis in obese rats was found to be related to its type in our previous studies [23,24], and whether the effects of different MCTs on glucose metabolism disorders in obesity are different is still unknown. Thus, the study aimed to investigate the effects and mechanisms of different types of MCTs on glucose metabolism disorders in obese rats.

## 2. Materials and Methods

### 2.1. Materials

The three triglycerides (CYT, CT, and LT) were prepared in our laboratory (chemical esterification, purity ≥ 99%) [23]. The biochemical assay kits for total triglyceride (TG), total cholesterol (TC), high-density lipoprotein (HDL)-c, low-density lipoprotein (LDL)-c, glucose, and insulin were purchased from Jiancheng Co. (Nanjing, China). The interleukin (IL)-4, IL-1β, and tumor necrosis factor (TNF) α were from ThermoFisher Scientific Co. (San Diego, CA, USA).

### 2.2. Animals and Experimental Design

A total 46 male Sprague–Dawley (SD) rats were purchased from Hubei Provincial Laboratory Animal Public Service Center (Wuhan, China). Ethical approval for all animal experimental procedures was granted by the Ethics Committee of Nanchang University, and the study adhered to the guidelines outlined in the Guidance for the Care and Use of Laboratory Animals (code: IACUC-20230303004, date: 3 March 2023). Following one week of adaptive feeding, the rats were randomly assigned to two groups: a control group (n = 6) and a high-fat-diet group (HFD, n = 40). The HFD group received a lard-rich fatty diet (based on D12451), while the control group was given a standard feed for a duration of 12 weeks. Subsequently, obese rats (n = 30) in the HFD group (body weight ≥ 115% control group) were randomly distributed into five intervention groups based on their body weight: HF group (n = 6), CYT group (n = 6), CT group (n = 6), LT group (n = 6), and high-fat restoring group (HR, n = 6). During the trial, the HF group continued with a high-fat diet, and the HR group were fed a standard base maintenance feed (AIN-93M, low-calorie diet), while the other groups received specially formulated feeds (CYT group—customized feed 1, CT group—customized feed 2, LT group—customized feed 3), wherein lard was entirely replaced by CYT, CT, and LT, respectively. Additional details, including the experimental design and feed formulas, can be found in the Supplementary Materials (Figure S1, Tables S1 and S2).

The dietary intervention trial lasted for 12 weeks, with rats housed three per cage under controlled environmental conditions: room temperature (25 ± 2 °C), humidity (40–60%), and a lighting cycle of 12 h on and 12 h off. Throughout the experiment, both diet and water were provided ad libitum.

### 2.3. Oral Glucose Tolerance Tests (OGTT)

To evaluate the glycemic activity of different MCTs, the OGTT was conducted at the end of dietary intervention. The obese rats were gavaged with glucose solution (40%, 2 g/kg BW) after overnight fasting (8 p.m.–8 a.m.), then blood samples (~100 μL) were collected from the tail vein of rats at appropriate intervals (0, 30, 60, 90, and 120 min), and blood glucose levels were measured using a glucometer, and the area under the curve (AUC) was also calculated.

### 2.4. Sample Collecting

At the end of the experiment, the rats were fasted for 12 h, then the rats were anesthetized with chloralhydrate and sacrificed through heart blood sampling. The blood samples were collected according to our previous method [25]. Then, the liver and abdominal adipose tissue were carefully removed, weighted, washed, and divided into appropriate volumes. Abdominal fat coefficient was calculated as [abdominal fat (g)/body weight (g) * 100] according to our previous work [25]. One part of the liver and adipose tissue was fixed in 4% paraformaldehyde for histological observation, and the rest of the liver and adipose tissue was immediately frozen in liquid nitrogen and stored at −80 °C for further use.

### 2.5. Biochemical Analysis

The serum lipids (TG, TC, HDL-c, and LDL-c) and glucose level were measured using a Leidu Chemray 240 automated analysis system (Shenzhen, China). The contents of serum insulin, inflammatory markers (IL-4, IL-1β, and TNF-α), and adipokine (adiponectin (ADP), leptin (LEP)) were determined using ELISA kits in accordance with the manufacturer’s instructions. The insulin resistance (HOMA-IR) was calculated as [FBG (mmol/L) * FINS (mIU/L)]/22.5, and insulin sensitivity index (ISI) was calculated as 1/(FBG (mmol/L) * FINS (mIU/L)) [26].

### 2.6. H&E and ORO Staining

Regarding the H&E staining procedure, the fixed liver and adipose tissue were embedded in paraffin and cut into 10 μm thin layers, then dewaxed with xylene and alcohol and stained with hematoxylin and eosin. Regarding the Oil Red O staining procedure, the immobilized hepatic and adipose tissues were cryo-sectioned into slices of 10 μm in thickness. Afterward, these sections underwent a sequence of washing, rinsing, and staining with Oil Red O dye. Observations of all the prepared slides were conducted through a microscope (Nikon E100, Tokyo, Japan), and photomicrographs were captured at 200× magnification.

### 2.7. Western Blotting

Liver and adipose tissue were selected to extract protein, and a series of protease inhibitors and phosphorylation protease inhibitors were added in this process. The protein concentration of the sample was determined, and the proteins were diluted to the same concentration and mixed with an appropriate amount of loading buffer, and finally heated for denaturation. The samples were separated using 10% or 12% gels and electroblotted onto PVDF membranes, then the membranes were blocked and washed with primary antibodies. After an overnight incubation at 4 °C, they were developed with ECL solution. Finally, the blots were visualized using a gel imaging system (Bio-Rad, Hercules, CA, USA). More primary antibody information, such as brand, species, and dilution ratio, can be found in the Supplementary Materials (Table S3).

### 2.8. Statistical Analysis

Results are expressed as the means ± standard deviations (SDs). All the data were analyzed via one-way ANOVA, followed by Tukey’s test using SPSS (version 22.0), and *p*-values < 0.05 were considered statistically significant.

## 3. Results

### 3.1. MCT Supplementation Suppresses Increases in BW, Plasma Lipids, and Lipid Deposition in Liver and Adipose Tissue

**Figure 1:**
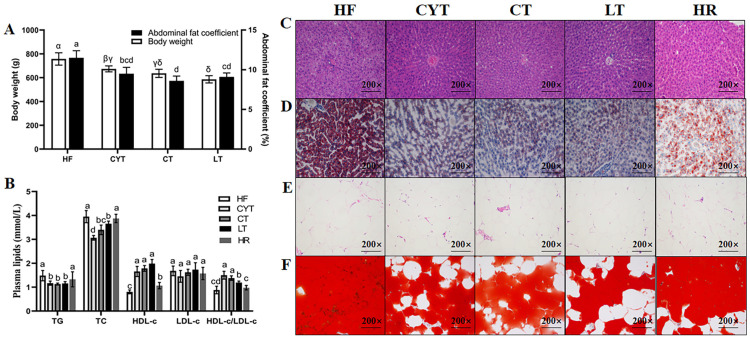
MCT intake improves obesity phenotype in rats. (**A**) Body weight and abdominal fat coefficient; (**B**) plasma lipid levels; (**C**,**E**) H & E staining of liver and adipose tissue; (**D**,**F**) Oil Red O staining of liver and adipose tissue; α, β, γ, δ or abcd means significant difference, *p* < 0.05.

At the end of the experiment (Figure 1), it was observed that the rats in the MCT groups exhibited significantly lower body weight compared to those in the HF group (*p* < 0.05). Furthermore, among the MCT groups, the CYT group demonstrated the highest body weight, followed by the CT and LT groups. The body weight of the HR group was found to be lower than that of the high-fat group; however, no statistically significant difference in abdominal fat coefficient was observed between the two groups. The MCT intervention also lowered the plasma lipid level in the obese rats, with no difference between the MCT groups. Furthermore, the MCT groups had smaller lipid droplets than that of the HF group both in the liver and adipose tissue. There were no significant differences observed in the extent of lipid deposition in the plasma and liver between the HF and HR groups.

### 3.2. MCT Improved Glucose Intolerance in Obese Rats

**Figure 2:**
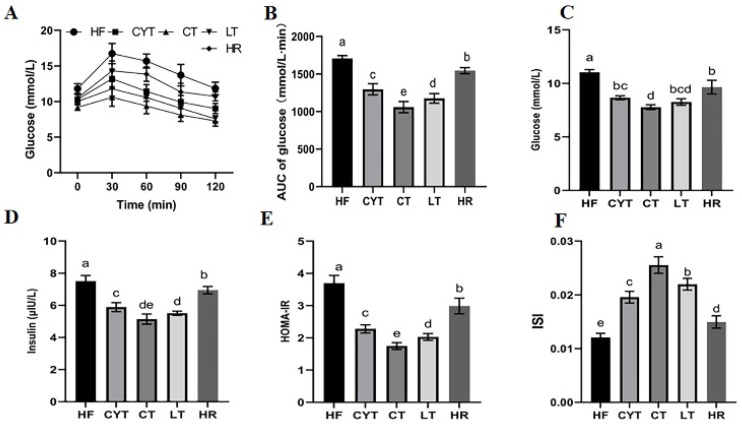
MCT intake improves glucose intolerance in obese rats. (**A**) Glucose levels during the OGTT; (**B**) AUC of the glucose levels during the OGTT; (**C**) the glucose levels in plasma after sacrifice; (**D**) the insulin levels in plasma at the end of the experiment; (**E**) HOMA-IR; (**F**) ISI. a–e means significant difference, *p* < 0.05.

For OGTT, the patterns in blood glucose levels in different groups were similar (Figure 2A). They all increased rapidly and reached the peaks at 30 min, and then declined to varying degrees with time. The HF group had a significantly higher blood glucose level than those of the MCT groups at different sampling points, with the highest AUC among all the groups (Figure 2B). In the MCT group, rats in the CYT group exhibited the highest blood glucose levels. Although no statistically significant difference was observed in blood glucose levels between the LT and CT groups, it is noteworthy that the CT group displayed comparatively lower blood glucose levels among all three groups. Additionally, similar to the blood glucose results, the CYT group had the highest AUC, followed by a decreasing trend in the LT and CT groups. Furthermore, the results for the insulin level were almost the same as for the blood glucose level; the HF group had the highest insulin level and HOMA -IR with the lowest ISI, while the CT group showed the lowest HOMA -IR and the highest ISI. The aforementioned parameters in the HR group exhibited lower values compared to those in the HF group; however, these findings still demonstrated higher levels when compared to the MCT groups.

### 3.3. MCT Treatment Lowered the Levels of Inflammatory Markers in Plasma

**Figure 3:**
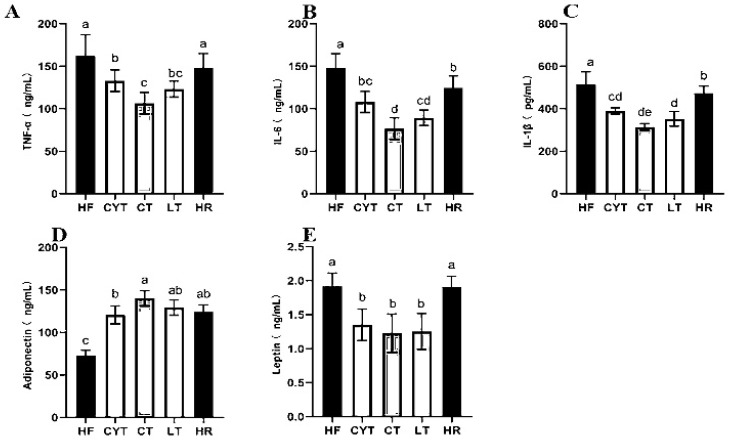
Effects of MCTs on plasma inflammatory factors and adipokines in obese rats. (**A**) TNF-α; (**B**) IL-6; (**C**) IL-1β; (**D**) adiponectin; (**E**) leptin. a–e means significant difference, *p* < 0.05.

The pathogenesis of obesity-related insulin resistance and T2DM involves a persistent low-grade inflammation [27]. In our study, the HF group had the highest levels of IL-6, TNF-α, and IL-1β among the groups, followed by the HR group. MCT treatment reduced the contents of plasma inflammatory markers compared to the rats fed with a high-fat diet (Figure 3A–C). In the MCT group, the CT group exhibited significantly lower levels of IL-6 and TNF-α compared to other groups. Conversely, the LT group demonstrated the lowest level of IL-1β. Notably, there was minimal disparity in the levels of these inflammatory factors between the LT and CT groups.

### 3.4. Effects of MCTs on Adipokines in Plasma and Adipose Tissue

A high-fat diet led to a disorder of adipokine secretion in adipose tissue, and MCT treatment modulated the adipokine secretion; the CT group had the highest adiponectin level both in plasma and adipose tissue among the MCT groups, followed by the LT and CYT groups, with no difference between the CT, HR, and LT groups (Figure 3D,E). A high-fat diet induced an elevation in leptin levels; the low-energy diet did not induce a decrease in leptin concentrations within the HR group. MCT treatment lowered leptin levels both in plasma and adipose tissue, with the CT group having the lowest leptin concentration, followed by the LT and CYT groups.

### 3.5. MCT Treatment Improved the Protein Expression Involved in Glucose Metabolism in the Liver

**Figure 4:**
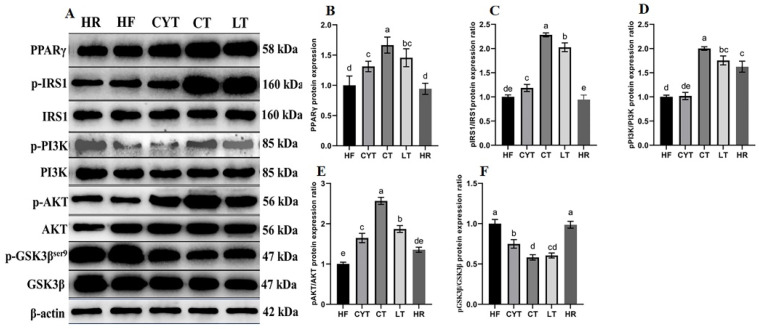
Effects of MCTs on the expression of the proteins involved in the IRS/PI3K/AKT signaling pathway in the liver. (**A**) Blots with molecular weights; (**B**) PPARγ; (**C**) IRS1 phosphorylation level; (**D**) PI3K phosphorylation level; (**E**) AKT phosphorylation level; (**F**) GSK3β phosphorylation level. a–e means significant difference, *p* < 0.05.

As shown in Figure 4, the expression and phosphorylation levels of IRS, PI3K, and AKT proteins in the MCT group exhibited varying degrees of growth compared to those in the HF group. In comparison to the HF group, HR exhibited only a marginal elevation in the expression levels of the aforementioned associated proteins. Notably, after MCT nutritional intervention, the CT group exhibited the highest activation level of the IRS/PI3K/AKT signaling pathway among obese rats, while a sequential decrease in activation was observed in the LT and CYT groups. The protein expression level of p-GSK3βSer9, a downstream protein of the PI3K/AKT pathway, was decreased through MCT treatment, with the CT group demonstrating the lowest expression level among the MCT groups. MCT treatment also decreased the protein expression of PEPCK and G6PC, which are upregulated through excessive calorie intake. The LT group exhibited the highest expression levels of these proteins, with no significant difference observed between the LT and CT groups.

**Figure 5:**
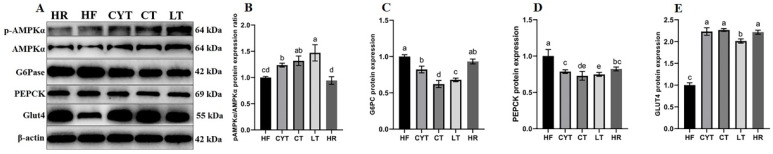
Effects of MCTs on the expression of the proteins involved in the AMPK signaling pathway in the liver. (**A**) Blots with molecular weights; (**B**) AMPKαphosphorylation level; (**C**) G6Pase; (**D**) PEPCK; (**E**) Glut4. a–e means significant difference, *p* < 0.05.

It has been reported that insulin sensitivity was associated with PPARγ activation [13]. In this study, we quantified the hepatic protein expression level of PPARγ. As illustrated in Figure 4, MCT intervention resulted in an upregulation of PPARγ expression in the liver, with the LT group exhibiting the highest protein expression among all MCT-treated groups. Notably, no significant difference was observed between the LT and CT groups. Additionally, MCT treatment led to an increase in the protein expression levels of AMPKα, pAMPK, and Glut4; notably, the CT group displayed the highest expression levels among all MCT-treated groups (Figure 5).

### 3.6. MCT Treatment Improved the Protein Expression Involved in Glucose Metabolism in Adipose Tissue

**Figure 6:**
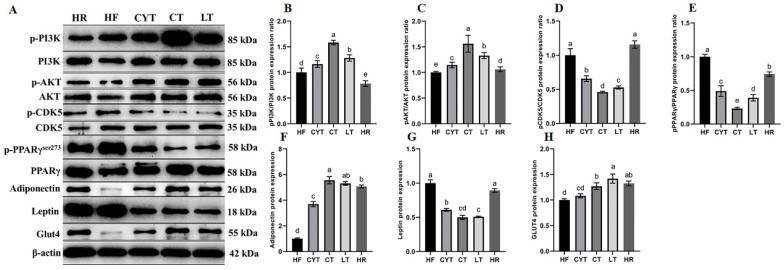
Effects of MCTs on the expression of the proteins involved in glucose metabolism in adipose tissue. (**A**) Blots with molecular weights; (**B**) PI3K phosphorylation level; (**C**) AKT phosphorylation level; (**D**) CDK5 phosphorylation level; (**E**) PPARγphosphorylation level; (**F**) adiponectin; (**G**) leptin; (**H**) Glut4. a–e means significant difference, *p* < 0.05.

The expression of proteins involved in the PI3K/AKT signaling pathway in adipose tissue was upregulated through MCT treatment, as depicted in Figure 6. Additionally, an increase in the expression of p-IRS and p-AKT was observed compared to rats fed a high- or low-energy diet (standard maintenance feed in HR group). The highest levels of these proteins were found in the CT group, followed by the LT and CYT groups. Furthermore, MCT intervention resulted in the upregulation of PPARγ expression in adipose tissue. The phosphorylation of PPARγ^ser273^ was lower in the CT group compared to the LT group, while the HF group exhibited the highest levels of PPARγ^ser273^ among all groups, followed by the HR group. Although the administration of MCTs had a limited impact on CDK5 protein expression, it significantly reduced the phosphorylation of CDK5. The CT group exhibited the lowest expression of pCDK5/CDK5, followed by the LT and CYT groups. In the MCT groups, there was no significant difference observed in pCDK5/CDK5 expression between the HF and HR groups. MCT and low-energy diet administration resulted in an upregulation of ADP protein expression in adipose tissue.

## 4. Discussion

In our previous work [24], we observed varying degrees of improvement in lipid metabolism in high-fat-diet-induced obese rats with the three different types of MCTs, and the efficacy of MCTs in enhancing lipid metabolism was found to be dependent on the specific MCFA composition. In this study, we further confirmed that MCT intervention reduced the lipid accumulation in the plasma, liver, and adipose tissue. Additionally, we observed a correlation between the specific types of MCFAs and their impact on enhancing glucose metabolism in high-fat-diet-induced obese rats.

MCTs have been identified in naturally occurring oils, such as palm kernel oil, coconut oil, and *Cinnamomum camphora* seed kernel oil. Additionally, milk fat serves as a source of MCTs [28,29]. Studies conducted on humans have reported that oils rich in MCTs and MCTs themselves can induce satiety and modulate proteins involved in lipid metabolism to ameliorate disorders related to lipid metabolism and weight gain [30]. Furthermore, MCTs have been utilized in the treatment of various neurological disorders like epilepsy and Alzheimer’s disease within the ketogenic diet [31].

At the beginning of the dietary intervention experiment, the HFD group exhibited significantly higher levels of BW, TG, TC HDL-c, and LDL-c compared to the control group (Figure S2). Following a 12-week MCT intervention, obese rats demonstrated decreased BW and blood lipid levels. Amongst the treatment groups, LT displayed the lowest BW and TG levels, followed by the CT and CYT groups, which aligns with our previous study [23]. Notably, the reduction in energy intake did not significantly ameliorate the obesity parameters in the obese rats, owing to the pre-existing dysregulation of lipid metabolism induced by high fat consumption (HR group). Given the well-established association between excessive energy intake and obesity, we administered a high-fat diet with a fat-to-energy ratio of 45% to induce obesity in rats. During the dietary-intervention phase, medium-chain triglycerides (MCTs) were incorporated as a full replacement for the lard component (m/m). Notably, even after this substitution, the custom feed remained a high-fat diet, and the daily energy intake of the rats still exceeded recommended levels. In our previous study, we also investigated whether lard intake is a cause of obesity. In our previous experiment, we set up a natural recovery group, in which we fed obese rats with basic maintenance feed (low-fat regular feed). The results showed that the food intake of the NR group was significantly higher than that of the natural control group, and there was almost no significant difference in obesity parameters between the NR group and the HF group [25]. An association between obesity and hyperglycemia, as well as insulin resistance, has been reported [32]. At the initiation of the dietary-intervention experiment, the HFD group exhibited a blood glucose level exceeding 10 mmol/L (Figure S2). Following various MCT interventions, a reduction in plasma glucose levels was observed in obese rats to varying extents. The glucose intolerance was also improved, and the MCT groups exhibited lower HOMA-IR and AUC of OGTT values, as well as higher ISI values compared to the HF group. Unfortunately, a low energy intake had a lesser effect on glucose intolerance in obese rats (Figure 2). Among the MCT groups, the CT group exhibited the most significant improvement in rats among these glucose tolerance parameters, followed by the LT and CYT groups. The findings suggest that MCTs can enhance glucose metabolism in obese rats, consistent with previous research by Geng et al., who reported the beneficial effects of MCTs on insulin resistance and inflammation in high-fat-diet-induced obese mice [33]. Moreover, the impact of MCTs on glucose metabolism in obese rats may be influenced by the specific type of MCT used, with CT demonstrating the most pronounced improvements in glucose metabolism among all tested types.

Multiple lines of preclinical and clinical research have substantiated the mechanistic association between chronic low-grade inflammation and overweight, as well as obesity [34]. The pro-inflammatory cytokine, TNF-α, frequently assumes a pivotal role in the pathogenesis of hepatic steatosis and insulin resistance (IR), thereby impeding insulin secretion and impairing β-cell function [35,36]. In this study, MCT intervention significantly reduced the TNF-α level in plasma. Similarly, the levels of IL-6 and IL-β were decreased via MCT intervention. The findings of this study suggest that MCTs may exert anti-inflammatory effects in obese rats. Furthermore, the results indicate that MCT intervention can effectively impede the progression of hyperglycemia and even T2DM in obese rats induced by a high-fat diet.

To elucidate the hypoglycemic mechanisms of MCTs in obese rats, we assessed the expression levels of insulin signaling-related proteins in both hepatic and adipose tissues. As a downstream signal factor of INS-R, IRS1 expression was increased through MCT treatment, and the phosphorylation level of IRS1 was upregulated as well. Moreover, the PI3K/AKT pathway was also activated in the MCT groups. The expression levels of p-PI3K/PI3K and p-AKT/AKT were significantly elevated in the MCT group compared to the HF group, while the CT group exhibited the highest activation of the PI3K/AKT pathway in both the liver and the adipose tissue. The PI3K/AKT pathway has been widely recognized for its pivotal role in the regulation of insulin resistance, glycogen synthesis, and gluconeogenesis, thereby maintaining glucose homeostasis in vivo [37,38]. Although a low energy intake has a certain effect on the PI3K/AKT pathway, the effect is far less than that of MCT treatment.

The liver, as the central organ in metabolism, plays an important role in glucose metabolism, and increasing the hepatic glycogen synthesis and lowering the hepatic gluconeogenesis can be helpful in restoring glucose homeostasis in vivo. Usually, the GSK-3β is active basally, and its kinase activity is inhibited when its N-terminal domain serine residue is phosphorylated by AKT under insulin stimulation. The activated GSK-3β can reduce the phosphorylation of GYS, resulting in the increased glycogenesis of hepatocytes [39]. In our work, MCT treatment lowered the expression of GSK-3β^ser9^, increasing glycogen synthesis in the liver. On the other hand, we also analyzed the hepatic glucose production in the liver. The protein expression levels of PEPCK and G6Pase, often be considered as rate-limiting enzymes in the glucose production process, were decreased after MCT intervention. MCT treatment lowered the glucose production in the liver.

High-fat-diet feeding can activate CDK5 in the adipose tissue, resulting in the phosphorylation of PPARγ at serine 273, a dominant that regulates the adipogenesis and fat cell gene expression [40]. Although the modification of PPARγ at ser 273 does not alter its adipogenic capacity, this change may decrease the expression of adiponectin, which is regarded as an insulin-sensitizing adipokine21. In this study, there were no different expression levels of CDK5 protein between the MCT groups and HF group, but MCT intervention did lower the phosphorylation of CDK5 and PPARγ at ser273. And the ADP was also increased both in the adipose tissue and plasma. It was also found that dihydromyricetin inhibited the phosphorylation of PPARγ^ser273^ through the ERK pathway, thereby increasing the secretion of ADP in adipose tissue and improving glucose metabolism without BW gain in diabetic fattened rats [41]. Moreover, MCT treatment upregulated AMPKα and pAMPKα protein in the adipose tissue, which can inhibit the CDK5/PPARγ pathway, and Fang et al. also found that activated AMPK can downregulate the CDK5/PPARγ pathway [42]. MCT intervention also increased the expression of Glut4, which has been considered an important protein in glucose transportation.

PPARγ has been a focus of intense research, as it is thought to be the master gene in not only regulating the differentiation of adipocytes, but also improving the glucose metabolism [43]. The activation of PPARγ has been reported to reduce inflammation, hyperglycemia, and insulin resistance and significantly improve glucose metabolism disorders in the body [44]. However, the classical PPARγ agonist rosiglitazone, a thiazolidinedione derivative, has been known to elicit action through full agonistic activity on PPARγ, which has had restricted use due to its side-effects of BW gain or lipogenesis in vivo [45,46]. Therefore, compounds that are the partial agonist or non-adipogenic agonist of PPARγ have been thought to be safer choices in the development of insulin sensitizer drugs. It has been reported that the fatty acids, including MCFAs and polyunsaturated fatty acid, are natural endogenous ligands of PPARγ, and MCFAs are regarded as the partial agonists of PPARγ [21,47,48]. In this study, MCT treatment did improve glucose metabolism without lipogenesis in obese rats.

**Figure 7:**
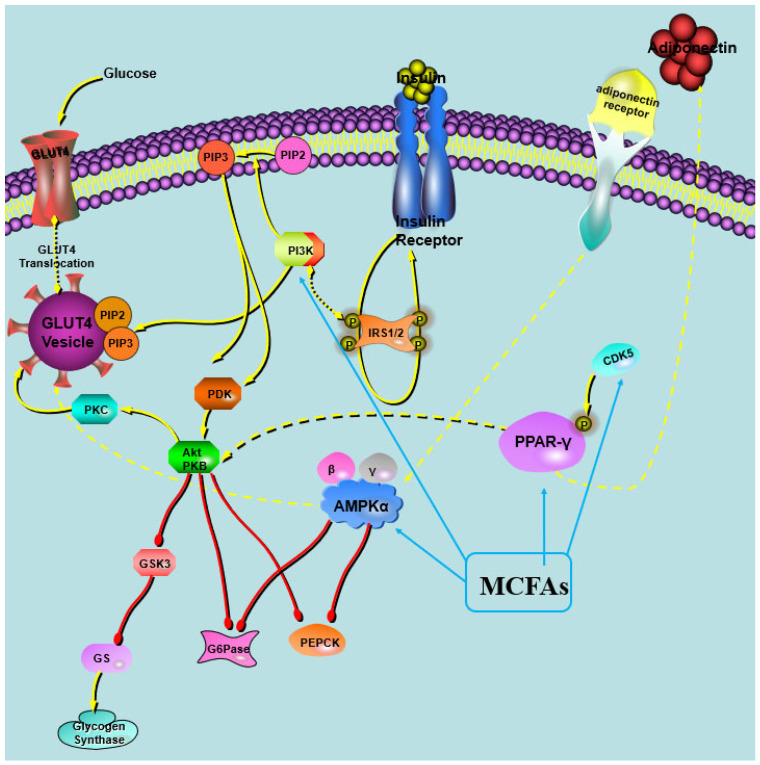
Illustration of the impact of MCT intervention on in vivo glucose metabolism.

For the partial activation of PPARγ by MCFAs, we speculate that MCTs may have a unique tripartite binding mode and different ligand binding domain (LBD) surface stability compared with other full PPARγ agonists. It was observed that the MCFAs within the LBP of PPARγ formed trimeric complexes, with each complex consisting of three identical copies of the ligand [21]. Both full and partial PPARγ agonists effectively stabilize the β-strand region of the PPARγ LBD, thereby significantly suppressing the CDK5-mediated phosphorylation of PPARγ [21]. These results were inconsistent with our finding that MCT intervention lowered the phosphorylation of PPARγ in the study. A putative hypothesis was proposed in the study (Figure 7). Moreover, it was found that the efficiency of MCFA to activate PPARγ was related to its type, with capric acid being the most efficient agonist, followed by lauric acid and caprylic acid [21,49]. Nonaka et al. reported that the dietary addition of capric triglycerides promoted GPR84-mediated GLP-1 secretion, thus increasing glucose tolerance and maintaining glucose homeostasis in mice [50]. These results suggest that capric acid had the most powerful effects on improving glucose metabolism among the MCFAs, and CT performed the best in maintaining glucose homeostasis. 

## 5. Conclusions

Our findings demonstrate that MCTs can partially activate PPARγ and inhibit the CDK5-mediated phosphorylation of PPARγser273, thereby ameliorating hyperglycemia and insulin resistance while restoring glucose homeostasis in obese rats. Additionally, this study confirms the differential effects of MCT types on improving glycolipid metabolism in obese rats, with CT exhibiting the greatest efficacy in alleviating glucose metabolism disorders, followed by LT and CYT. The limitation of the study was that the fatty acid profile of the lard was unknown, and the specific fatty acids would have a certain impact on the subsequent experimental results. Notably, these results diverge from previous experiments, where LT showed superior effects on lipid metabolism in obese rats, suggesting that each type of MCT may exert distinct influences on various metabolic disorders in obesity models, necessitating further investigation.

## Data Availability

The data presented in this study are available on reasonable request from the corresponding author.

## Supplementary Materials

The following supporting information can be downloaded at: https://www.mdpi.com/article/10.3390/foods13020241/s1, The animal experiment description (Figure S1), the initial glucose concentration in obese rats during the dietary-intervention phase (Figure S2), the composition and energy contents of diets (Tables S1 and S2), and the primary antibody details (Table S3) are provided in Supplementary Materials.
