# Supplementary material for: Effect of Different Medium-Chain Triglycerides on Glucose Metabolism in High-Fat-Diet Induced Obese Rats
Source: Foods. 2024 Jan 11;13(2):241. doi: 10.3390/foods13020241 (PMC10815142; doi:10.3390/foods13020241)
Supplement: Supplementary file 1 [file foods-13-00241-s001.zip › foods-2818587-supplementary.pdf]

Effect of different medium chain triglycerides on glucose metabolism in obese rats induced by high fat diet

Jiaheng Xia, Zhixin Wang, Ping Yu*, Xianghui Yan, Junxin Zhao, Guohua Zhang, Deming Gong, Zheling Zeng *

* Corresponding author: Professor Zheling Zeng, PhD

State Key Laboratory of Food Science and Technology, Nanchang University, Nanchang 330047, China

Jiangxi Province Key Laboratory of Edible and Medicinal Resources Exploitation, Nanchang University, Nanchang 330031, China

School of Resource and Environmental and Chemical Engineering, Nanchang University, Nanchang 330031, China

E-mail: zlzengjx@hotmail.com; zlzengjx@163.com

Tel.: +86-791-88510806 Fax: +86-791-8851080

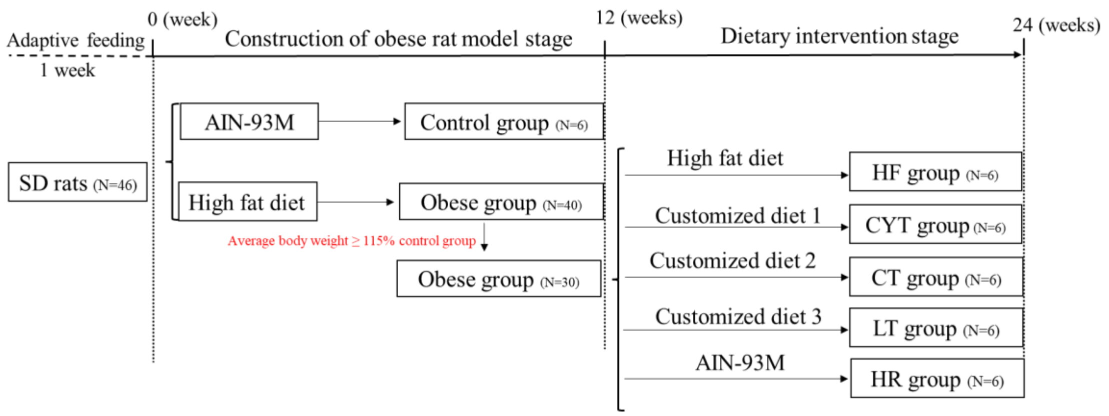

Figure S1 Description of the animal experiment

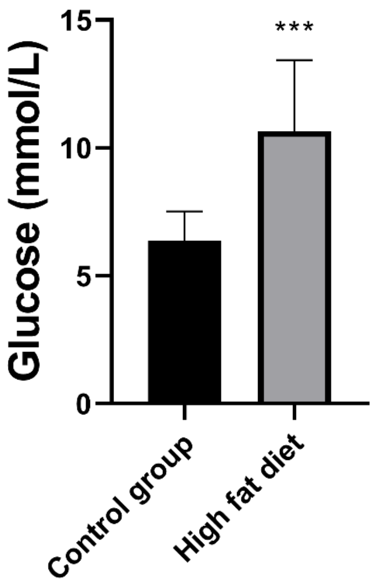

Figure S2 The concentration glucose in obese rats at the beginning of the dietary intervention phase (12w).

Table S1. Composition and Energy Contents of Diets.

Product	High fat diet		Custom feed 1		Custom feed 2		Custom feed 3	
	gm%	Kcal%	gm%	Kcal%	gm%	Kcal%	gm%	Kcal%
Protein	24	20	24	20	24	20	24	20
Carbohydrate	41	35	41	35	41	35	41	35
Fat	24	45	24	45	24	45	24	45
Ingredients	Gm	Kcal	Gm	Kcal	Gm	Kcal	Gm	Kcal
Casein	233.06	932.24	233.06	932.24	233.06	932.24	233.06	932.24
L-Cystine	3.50	14	3.50	14	3.50	14	3.50	14
Corn starch	84.83	339.32	84.83	339.32	84.83	339.32	84.83	339.32
Maltodextrin 10	116.53	466.12	116.53	466.12	116.53	466.12	116.53	466.12
Sucrose	201.36	805.44	201.36	805.44	201.36	805.44	201.36	805.44
Cellulose	58.26	-	58.26	-	58.26	-	58.26	-
Soybean oil	29.13	262.17	29.13	262.17	29.13	262.17	29.13	262.17
Lard	206.84	1861.56	-	-	-	-	-	-
Caprylic triglyceride			206.84	1861.56	-	-	-	-
Capric triglyceride			-	-	206.84	1861.56	-	-
Lauric triglyceride			-	-	-	-	206.84	1861.56
Mineral mix	11.65	-	11.65	-	11.65	-	11.65	-
Dicalcium phosphate	15.15	-	15.15	-	15.15	-	15.15	-
Calcium carbonate	6.41	-	6.41	-	6.41	-	6.41	-
Potassium citrate, 1 H_2O	19.23	-	19.23	-	19.23	-	19.23	-
Vitamin mix	11.56	46.24	11.56	46.24	11.56	46.24	11.56	46.24
Choline bitartrate	2.33	-	2.33	-	2.33	-	2.33	-
FD&C Red Dye #40	0.058	-	0.058	-	0.058	-	0.058	-
Total	1000	4727.09	1000	4727.09	1000	4727.09	1000	4727.09

Table S2. Composition and Energy Contents of Diets.

Product	Normal diet (AIN-93M)		High fat diet (D12451)		Custom feed	
	gm%	Kcal%	gm%	Kcal%	gm%	Kcal%
Protein	14.2	14.7	24	20	24	20
Carbohydrate	73.1	75.9	41	35	41	35
Fat	4	9.4	24	45	24	45
Ingredients	Gm	Kcal	Gm	Kcal	Gm	Kcal
Casein	140	560	233.06	932.24	233.06	932.24
L-Cystine	1.8	7.2	3.50	14	3.50	14
Corn starch	495.692	1983	84.83	339.32	84.83	339.32
Maltodextrin 10	125	500	116.53	466.12	116.53	466.12
Sucrose	100	400	201.36	805.44	201.36	805.44
Cellulose	50	-	58.26	-	58.26	-
Soybean oil	40	360	29.13	262.17	29.13	262.17
Lard	-	-	206.84	1861.56	-	-
Lauric triglyceride					206.84	1861.56
Mineral mix	35	-	11.65	-	11.65	-
Dicalcium phosphate	-	-	15.15	-	15.15	-
Calcium carbonate	-	-	6.41	-	6.41	-
Potassium citrate, 1 H_2O	-	-	19.23	-	19.23	-
Vitamin mix	10	40	11.56	46.24	11.56	46.24
Choline bitartrate	2.5	-	2.33	-	2.33	-
FD&C Red Dye #40	-		0.058	-	0.058	-
Total	1000	3850	1000	4727.09	1000	4727.09

Table S3 Primary Antibody Information

Antibodies	Details	Dilution
IRS1	Affinity, Rabbit Polyclonal, AF6273	1:1000
Phospho-IRS1 (Ser307)	Affinity, Rabbit Polyclonal, AF3272	1:1000
Phospho-PI3Kp85 (Tyr458)[Tyr467]/p55 (Tyr199)	Affinity, Rabbit Polyclonal, AF3242	1:1000
PI3K p85/p55	Affinity, Rabbit Polyclonal, AF6242	1:1000
Phospho-AKT1/2/3 (Ser473)	Affinity, Rabbit Polyclonal, AF0016	1:500
pan-AKT1/2/3	Affinity, Rabbit Polyclonal, 26513-1-AP	1:800
GSK3 beta	Affinity, Rabbit Polyclonal, AF5016	1:1000
Phospho-GSK3 beta (Ser9)	Affinity, Rabbit Polyclonal, AF2016	1:1000
AMPK alpha	Affinity, Rabbit Polyclonal, AF6423	1:800
Phospho-AMPK alpha (Thr172)	Affinity, Rabbit Polyclonal, AF3423	1:1000
PEPCK	Proteintech, Rabbit Polyclonal, 14892-1-AP	1:1000
G6PC	Proteintech, Rabbit Polyclonal, 22169-1-AP	1:1000
GLUT4	Proteintech, Mouse Monoclonal, 66846-1-Ig	1:1000
CDK5	Affinity, Mouse monoclonal, BF0121	1:10000
Phospho-CDK5 (Ser159)	Affinity, Rabbit Polyclonal, AF8366	1:1000
PPAR Gamma	Bioss, Rabbit Polyclonal, bs-4590R	1:1000
Phospho-PPAR Gamma (ser273)	Bioss, Rabbit Polyclonal, bs-4888R	1:1000
β-actin	Proteintech, Mouse Monoclonal,66009-1-Ig	1:10000
Goat anti- mouse	Proteintech, HRP conjugate, SA00001-1	1:5000
Goat Anti-Rabbit	Proteintech, HRP conjugate, SA00001-2	1:5000
